# MCI-frcnn: A deep learning method for topological micro-domain boundary detection

**DOI:** 10.3389/fcell.2022.1050769

**Published:** 2022-11-30

**Authors:** Simon Zhongyuan Tian, Pengfei Yin, Kai Jing, Yang Yang, Yewen Xu, Guangyu Huang, Duo Ning, Melissa J. Fullwood, Meizhen Zheng

**Affiliations:** ^1^ Shenzhen Key Laboratory of Gene Regulation and Systems Biology, School of Life Sciences, Southern University of Science and Technology, Shenzhen, China; ^2^ School of Biological Sciences, Nanyang Technological University, Singapore, Singapore; ^3^ Cancer Science Institute of Singapore, National University of Singapore, Singapore, Singapore; ^4^ Institute of Molecular and Cell Biology, Agency for Science, Technology and Research (A*STAR), Singapore, Singapore

**Keywords:** deep learning, topological micro-domain, faster R-CNN algorithm, 3D genome organization, domain boundary

## Abstract

Chromatin structural domains, or topologically associated domains (TADs), are a general organizing principle in chromatin biology. RNA polymerase II (RNAPII) mediates multiple chromatin interactive loops, tethering together as RNAPII-associated chromatin interaction domains (RAIDs) to offer a framework for gene regulation. RAID and TAD alterations have been found to be associated with diseases. They can be further dissected as micro-domains (micro-TADs and micro-RAIDs) by clustering single-molecule chromatin-interactive complexes from next-generation three-dimensional (3D) genome techniques, such as ChIA-Drop. Currently, there are few tools available for micro-domain boundary identification. In this work, we developed the MCI-frcnn deep learning method to train a Faster Region-based Convolutional Neural Network (Faster R-CNN) for micro-domain boundary detection. At the training phase in MCI-frcnn, 50 images of RAIDs from *Drosophila* RNAPII ChIA-Drop data, containing 261 micro-RAIDs with ground truth boundaries, were trained for 7 days. Using this well-trained MCI-frcnn, we detected micro-RAID boundaries for the input new images, with a fast speed (5.26 fps), high recognition accuracy (AUROC = 0.85, mAP = 0.69), and high boundary region quantification (genomic IoU = 76%). We further applied MCI-frcnn to detect human micro-TADs boundaries using human GM12878 SPRITE data and obtained a high region quantification score (mean gIoU = 85%). In all, the MCI-frcnn deep learning method which we developed in this work is a general tool for micro-domain boundary detection.

## Introduction

In eukaryotic nuclei, chromatin is organized into three-dimensional (3D) conformation with multi-scale and is essential for gene transcription. By application of ChIA-PET (chromatin interaction analysis by paired-end tag sequencing) strategy (Fullwood et al., 2009), it has been suggested that CTCF (CCCTC-binding factor)-mediated chromatin interaction anchors tethered together as foci interacting with RNAPII (RNA polymerase II) by selectively drawing specific genes for coordinated transcription (Li et al., 2012; Tang et al., 2015). These loops often interconnect as a daisy-chain-like structure, where CTCF-mediated chromatin contact domains (CCDs) are highly consistent with topologically associated domains (TADs) identified by Hi-C (high-throughput chromosome conformation capture) (Lieberman-Aiden et al., 2009; Dixon et al., 2012; Nora et al., 2012), RNAPII-associated interaction domains (RAIDs) are corresponding to transcription factories (Li et al., 2012; Zheng et al., 2019). Dysregulations in chromatin interaction loops or chromatin structural domains have been found to be associated with certain diseases such as cancer (Li et al., 2012; Krijger and de Laat, 2016; Wang et al., 2020).

The emerging ligation-free 3D genome techniques for the identification of multiplex chromatin interactions lead the chromatin structures of CCDs, TADs, and RAIDs to a high-resolution sub-domain level to reveal novel aspects of chromatin organization. These include split-pool recognition of interactions by tag extension (SPRITE) data, which indicates that chromatin is separated into discrete contact hubs (Quinodoz et al., 2018). By chromatin-interaction analysis *via* droplet-based and barcode-linked sequencing (ChIA-Drop) data (Zheng et al., 2019), we uncovered chromatin contacts involving multi-way contacts that covered different TADs or fall within a single TAD and clustered into micro-domains with some similarities of interacting features, which can be visualized directly by MCIBox, a toolkit for single-molecule multi-way chromatin interaction visualization and micro-domain identification (Tian et al., 2022). Previous analysis has shown that the micro-domains own a distinctive signature of transcription activity, while their boundary detection has yet to be thoroughly studied. Although there are more than 20 kinds of methods for domain boundary calling, they are based on chromatin contacts’ pileup coverage (Zufferey et al., 2018). Here, we introduce an intuitive method to define the boundaries of the micro-domains.

Object detection is a key branch of computer vision technologies, which aims to use computers to scan and identify the instances and their locations by mimicking the human visual system—“What You See Is What You Get.” One deep-learning based object detection algorithm, Faster R-CNN (Faster Region-based Convolutional Neural Network) (Ren et al., 2015), shares convolutional features using an attention mechanism between region proposal networks (RPNs) and Fast R-CNN detectors. Due to their good performance in terms of detection accuracy and speed, Faster R-CNN Detectors have been widely used in many areas, such as self-driving (Agarwal et al., 2019), face detection (Zhan et al., 2016; Sabir et al., 2022), and disease detection (Ma et al., 2020). In this study, we set up a new program by adopting Faster R-CNN algorithm to detect multi-way chromatin interaction clustered micro-domains, termed MCI-frcnn. The results show that a well-trained MCI-frcnn can detect the micro-domain boundary rapidly (∼19 ms/image) and with high accuracy of assessment at the genomic Intersection over Union (gIoU) (more than 75%) for RAIDs and TADs of *Drosophila* and human data.

## Methods and results

**FIGURE 1:**
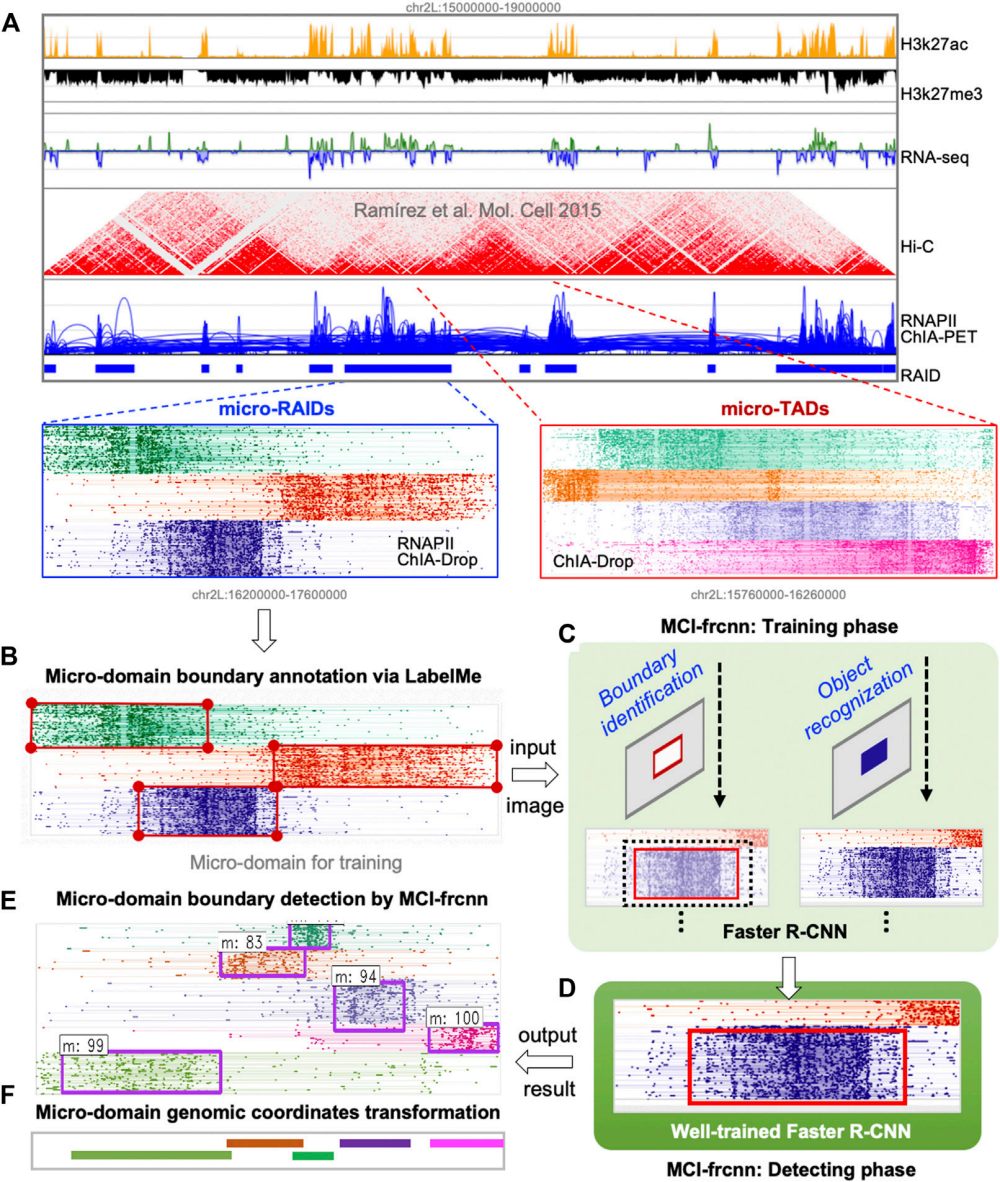
Scheme of MCI-frcnn. **(A)** Using the clustering algorithm based visualization tool MCIBox, we dissected RNAPII-associated interaction domains (RAIDs) from ChIA-PET data into micro-RAIDs by RNAPII-enriched ChIA-Drop data, and dissolved topologically associated domains (TADs) from Hi-C data into micro-TADs by ChIA-Drop data. **(B)** An image of a RAID with clustered fragment-view of micro-domains for the MCI-frcnn training set is subjected to LabelMe annotation tool to draw their ground truth boundary boxes. **(C)** MCI-frcnn trains Faster R-CNN networks using a training set for boundary identification and recognition of a micro-domain. **(D)** After a number of epochs (iterations) of training and finetuning, the Faster R-CNN networks are trained and ready for detection. **(E)** Boundaries (bounding boxes) of micro-domains detected by MCI-frcnn from new images of the detecting set, with a detected class name and a classification score. **(F)** Micro-domain genomic coordinates transformed from pixel boundary boxes.

**FIGURE 2:**
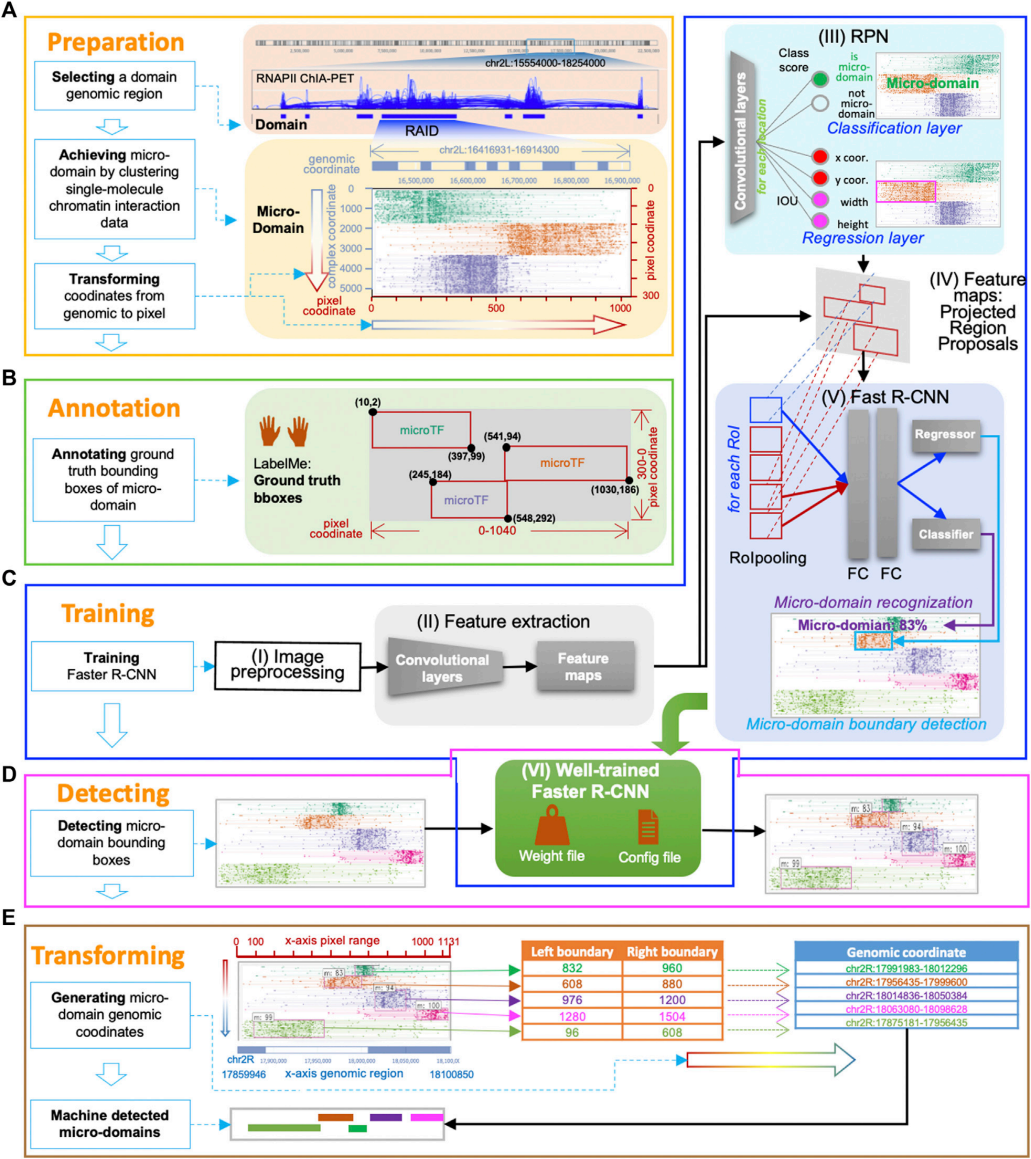
MCI-frcnn working flow. **(A)** Data preparation phase. **(B)** Annotation phase. **(C)** Training phase includes these steps: the image preprocessing step (I), the feature extraction step (II), the RPN training step (III), the Regions of Interest (RoIs) projection step (IV), the Fast R-CNN detector training step (V), and the finetuned Faster R-CNN step (VI). **(D)** Detecting phase. **(E)** Micro-domain genomic coordinates transforming phase.

We applied the Faster R-CNN algorithm to develop MCI-frcnn, a deep learning based tool to detect boundaries of micro-domains robotically. MCI-frcnn includes five phases: data preparing phase, annotation phase, training phase, detecting phase, and micro-domain genomic coordinates transforming phase (Figures 1, 2). Scripts of MCI-frcnn is available at the public repository GitHub (https://github.com/ZhengMZLab/MCI-frcnn).

### Data preparing phase

In the data preparation phase, our main task is to prepare input images for Faster R-CNN. Using MCIBox clustering-based visualization tool (Tian et al., 2022), we observed that single-molecule multi-way chromatin interaction complexes within a single TAD or RAID can be clustered into different groups of micro-domains. For example, we can identify micro-TADs by decomposing a TAD structure *via* ChIA-Drop data, as well as obtain several micro-RAIDs by dissecting a RAID *via* RNAPII ChIA-Drop data (Figures 1A, 2A).

Micro-TADs and micro-RAIDs are displayed as clustered fragment-views by MCIBox, in which the fragments of each chromatin complex are displayed in their linear genomic alignments along the *x*-axis, and the different single-molecule complexes are arranged along the *y*-axis. After we obtained a proper clustered Fragment-view of a RAID in MCIBox, we stored the view as a PNG file, accompanied by the genomic coordinate of the RAID. All these images were divided into two sets: a training set and a detection set (i.e., test set), and the training set were also used as the validation set to perform cross-validation.

### Annotation phase

In the annotation phase, our main task was to mark the micro-domain ground truth boundary box (i.e., bounding box). Using the interactive interface of the annotation software, LabelMe, we manually drew a rectangular enclosed line for each micro-domain as its ground truth boundary in each training image, and LabelMe identified the pixel size of the whole image and recorded the pixel coordinates of each micro-domain bounding box (Figures 1B, 2B). These annotations for all images in the training set were then collected into a unique document, in which each row represents one piece of micro-domain information including image path, pixel coordinates of the left-top point and the right-bottom point of the micro-domain’s bounding box, and the category of the micro-domain. In this study, there is only one category, which is micro-domain (*m*).

### Training phase

#### Procedures in the training phase

In the training phase, our main task was to train a Faster R-CNN for micro-RAID recognition. Faster R-CNN consists of two major modules, RPN (region proposal network) is a convolutional network to generate the region proposals, and Fast R-CNN (Fast Region-based Convolutional Neural Network) is a detector network. This phase included the following steps (Figures 1C, 2C): the image preprocessing step (I), the feature extraction step (II), the RPN training step (III), the regions of interest (RoIs) projection step (IV), the Fast R-CNN detector training step (V), and the finetuned Faster R-CNN step (VI).

#### Training step (I-II): Image preprocessing and feature extraction

The purpose of the image preprocessing step (I) was to rescale the training image to a predefined size and the micro-domain bounding boxes' pixel coordinates accordingly. The goal of the feature extraction step (II) was to feed the preprocessed image of a domain into the backbone convolutional neural network to calculate feature maps.

#### Training step (III): Training RPN

The RPN training step (III) aims to find whether there is a micro-domain existing in the domain and to find its boundary proposals, which refer to a set of rectangular bounding boxes generated by RPN, that highly overlapped with ground truth bounding boxes of micro-domains. In detail, the RPN training step includes the following procedure: first, RPN generates many fixed-size anchor boxes that can evenly cover the entire image; second, the features mapped out by the extraction module are passed into a convolutional network, and the following two sibling (parallel) convolution layers for classification and regression, respectively. The classification layer seeks to determine if an anchor box consists of a micro-domain (foreground) or not (background) and gives out two classification possibility scores *via* the *softmax* function. The regression layer is used for boundary box regression, which produces four regression coefficients of each of the anchor boxes for each pixel in the feature map.

Next, the anchors with high classification scores are subjected to the calculation of the intersection over union (IoU) value with ground truth boundary boxes of a micro-domain. Following this, the anchors with higher IoU scores are classified as candidate boundaries of micro-domains. Furthermore, a certain number of micro-domains are randomly selected from individual images as a mini-batch. For every mini-batch, in order to assess the extent of the match between the RPN detected boundary and ground truth boundary of a micro-domain, the RPN loss functions (*rpn_loss = loss_rpn_regression + loss_rpn_classifier*) are obtained by using the *smooth*_*L1*_ and *softmax* functions, respectively.

#### Training step (IV): RoI projection

In the next step, the function of the RoI projection step (IV) is to export RPN-selected proposals (RoIs) as training samples, by projecting each proposal (candidate boundary box) from the feature maps to the Fast R-CNN detector for RoI pooling operation, which functions to give a fixed size feature map to meet the requirement of the following two fully connected layers.

#### Training step (V): Fast R-CNN detector training

The purpose of the Fast R-CNN detector training step (V) is to perform further classification and boundary location adjustment based on every RoI from the RPN. First, each proposal derived from an RPN uses the RoI pooling technique to normalize them into feature maps of the same size and one-dimensional feature vectors. Then, the feature vector is sent to the following two fully connected layers for learning. The learned features are then sent to the subsequent component classifier (*softmax*) and regressor (bounding box regression) for micro-domain classification recognition and boundary finetuning, and to generate Fast R-CNN Detector loss functions (*fastrcnn_loss = loss_detector_classifier + loss_detector_regression*) for backpropagate parameters.

#### Training step (VI): Finetuned faster R-CNN

The finetune Faster R-CNN step (VI) improves the accuracy of the learning machine by backpropagating parameters of the current training epoch (iteration) to the learning machine, if the current total loss (*total_loss = rpn_loss + fastrcnn_loss*) is smaller than the average. These backpropagate parameters are saved in weight files (e.g., *model_frcnn.hdf5*), and the configuration information is stored in a configuration file (e.g., *config.pickle*).

With each epoch of training, the loss curve continues to drop, the accuracy curve continues to increase, and the parameters are constantly updated. When the loss curve approaches a stable value near 0, and the accuracy curve approaches a stable value near 1, we consider that the Faster R-CNN model for micro-domain detection is trained and ready for new micro-domain detection (Figures 1D, 2C).

### Detecting phase

In the detecting phase, our goal is to use the trained Faster R-CNN to detect micro-domains from new images. In the data preparation phase, we prepared new domain images for Faster R-CNN to detect micro-domain boundaries (Figures 1D, 2D). After inputting the waiting-detection images into the trained model, the micro-domain detection process is begun. The final step of this detection process is directly outputting the testing results, instead of backpropagating parameters for finetuning the machine during the training process. The detecting phase is relatively rapid, we can recognize all micro-domains in an entire image within 20 ms. Thus, in this phase, we can obtain the final detection results of MCI-frcnn, which includes micro-domain boundary classification scores (the probability of being recognized as a micro-domain) and boundary box coordinates (the occupation area of the micro-domain), as shown in Figures 1E, 2D.

### Micro-domain genomic coordinate transforming phase

**FIGURE 3:**
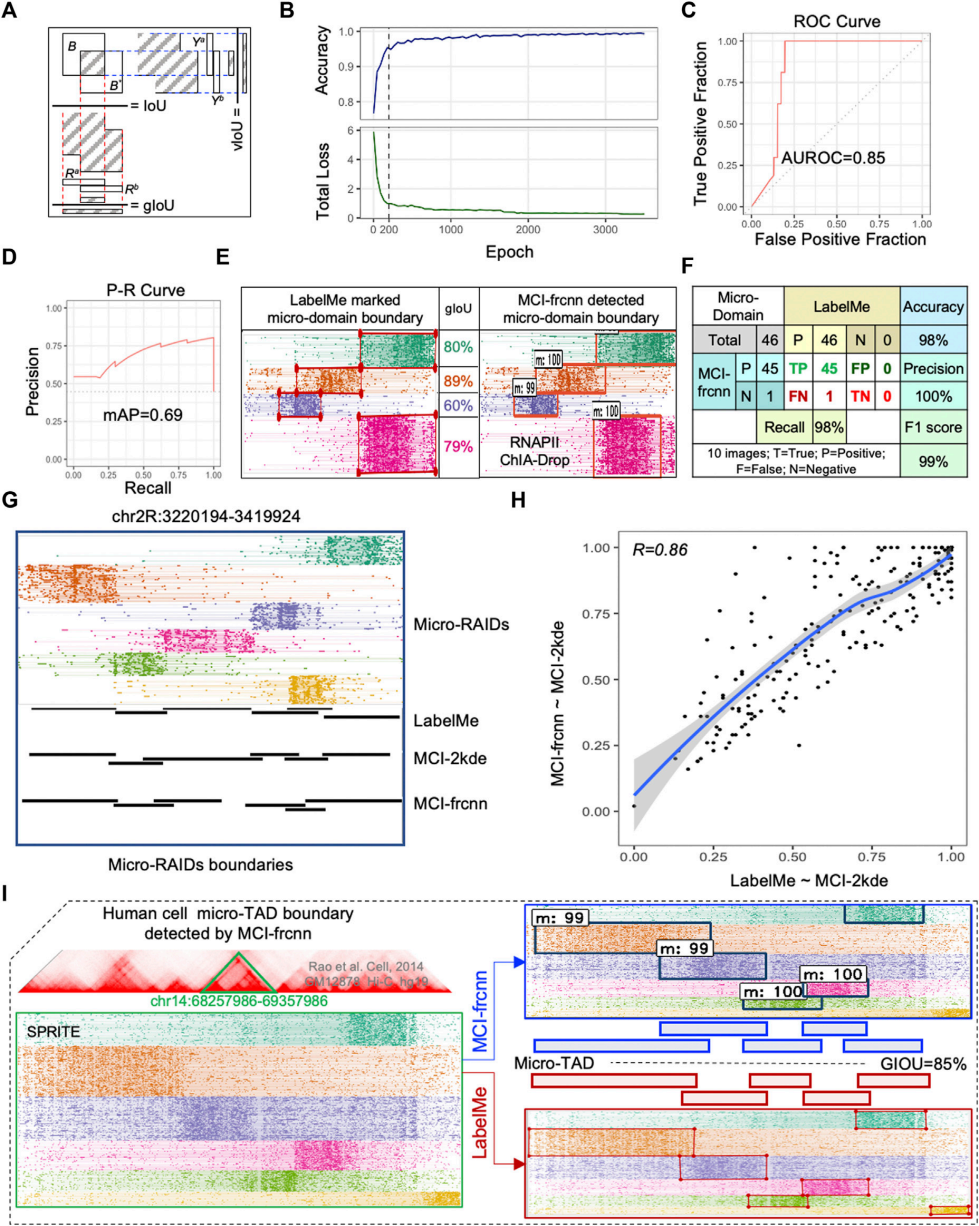
MCI-frcnn Performance. **(A)** Illustration of values of the intersection over union value (IoU), genomic IoU (gIoU), and vertical IoU (vIoU). **(B)** Curves of classifier accuracy for bounding boxes from RPN, and the total loss value of Faster R-CNN (*total_loss = loss_rpn_regression + loss_rpn_classifier + loss_detector_classifier + loss_detector_regression*). **(C)** Receiver operator characteristic (ROC) curve and the value of area under ROC curve (AUROC). **(D)** Precision-Recall (P-R) curve and mean average precision value (mAP). **(E)** Example of micro-domains genomic boundaries detected by MCI-frcnn machine, together with gIoUs when compared with their ground truth genomic regions. **(F)** Errors obtained from the 46 micro-RAIDs from the 10 testing images listed in Figure 4, including values of accuracy, precision, recall, and F1 score, by comparing MCI-frcnn with ground truth. **(G)** Screenshot of micro-RAIDs at chr2R:3220194-3419924 with track of micro-RAIDs in fragment view, following by domain view defined by LabelMe, MCI-2kde, and MCI-frcnn programs. **(H)** Scatterplot presents the correlation of intersect region from MCI-2kde versus LabelMe to MCI-2kde versus MCI-frcnn. Pearson correlation coefficient (R) value is shown. **(I)** Example of micro-TAD boundary identified using MCI-frcnn on SPRITE data from the human GM12878 cell line. Left panel presents the screenshot from MCIBox of the micro-TADs at chr14:68257986-69357986 (bottom), which is zoomed-in from TADs (up); right panel indicates the boundaries detected by MCI-frcnn and LabelMe, m:# (such as m:99) represents micro-domain: detectable percentage.

The function of the micro-domain genomic coordinate-transforming phase is to identify micro-domain genomic coordinates according to their pixel boundary boxes detected by Faster R-CNN (Figures 1F, 2E). First, if there are multiple detected boundary boxes that cover one micro-domain area with vertical IoU (vIoU) (Figure 3A; Supplementary Material) more than 80%, they become merged. Next, the coordinates of the most left and right points of the bounding box are transformed into genomic coordinates and regarded as the micro-domain’s genomic boundary. With this, MCI-frcnn finally defines micro-domain boundaries.

### Applied MCI-frcnn for micro-RAID boundary detection

We prepared 50 images of RAIDs by MCIBox on RNAPII ChIA-Drop data from *Drosophila melanogaster* S2 cell line as the Faster R-CNN training set, obtaining 261 micro-RAIDs in total. Then, we drew ground truth bounding boxes for each of the 261 micro-RAIDs, using LabelMe.

Before the training phase, we performed a 5-fold cross-validation on the same training set of the 50-RAID images, training 300 epochs of each group independently. To evaluate the detection efficiency of the micro-RAID boundary, we used a new coefficient: genomic intersection over union (gIoU), which calculates the ratio of the genomic length of the overlapping region over the whole union of the two genomic regions (Supplementary Material). This reflects the similarity between two micro-domains from MCI-frcnn and ground truth (Figure 3A). The 5-fold cross-validation results show that the mean gIoU value of each validation group is 76.2% (79%, 70%, 77%, 79%, and 76%, individually), which indicates that MCI-frcnn shows better generalization ability in adapting to new samples.

Subsequently, these images of the training set were subjected to training. We found both the accuracy curve and the loss line reached a stable phase after ∼200 epochs of training by running about 11 h (Figure 3B). Theoretically, the Faster R-CNN implemented here is sufficiently trained for testing. However, the ultimate criterion for evaluating the quality of a learning machine is its ability to identify micro-domain boundaries, and we found the detection results are still not sufficient for the beginning of the stable phase. To obtain a more accurate detector, we continuously trained the machine for a longer time. Until 3,500 epochs (∼170 h, i.e., ∼7 days), we did not detect any more obvious changes occurring in the loss curve, suggesting that we had obtained a well-trained Faster R-CNN detector (Figure 3B).

**FIGURE 4:**
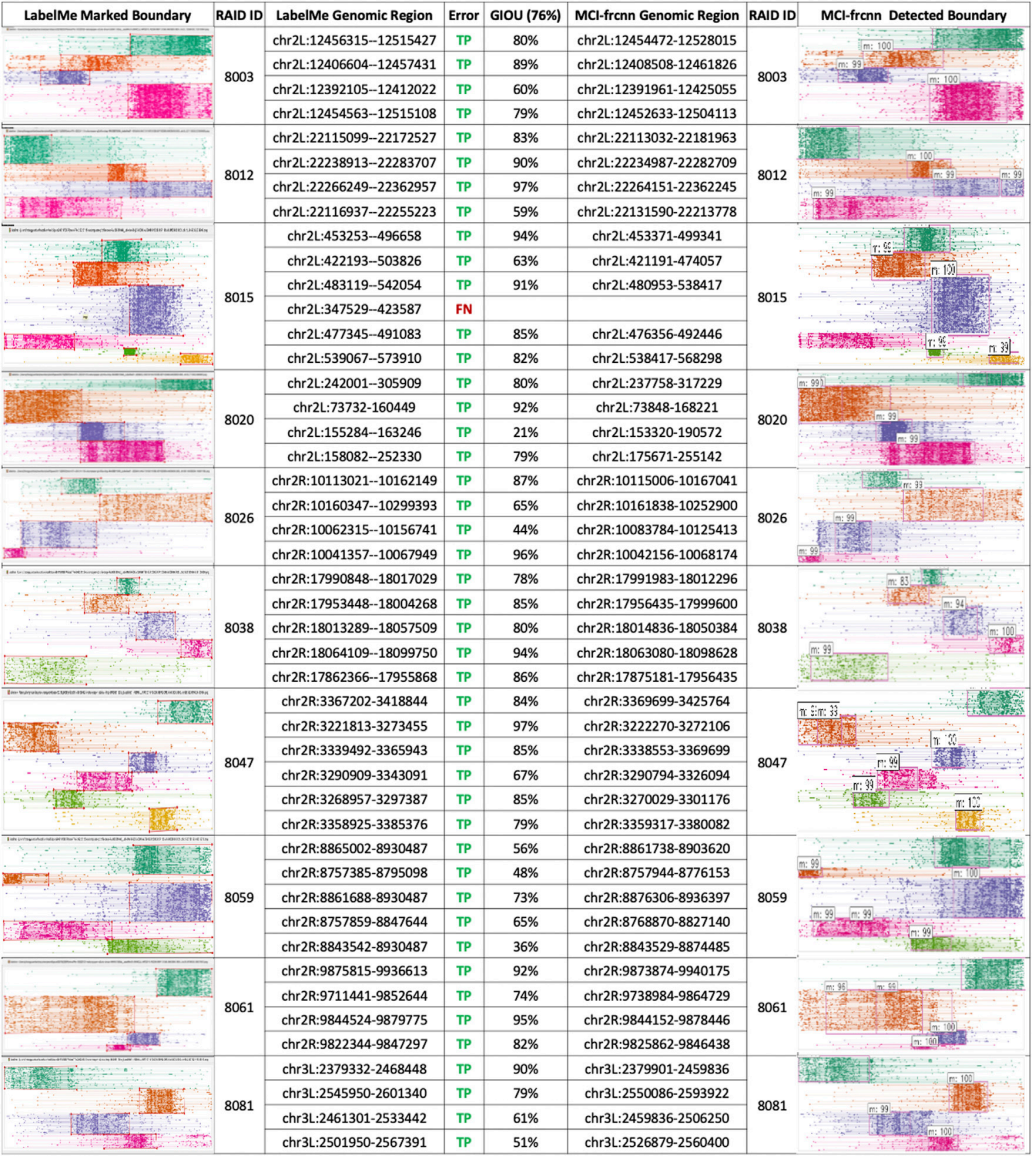
MCI-frcnn detection results. Table of the results of micro-domain boundaries detected from the 10 new RAID clustering images using MCI-frcnn (right side), and their corresponding ground truth boundaries on the left-side. gIoU and error type of each micro-domains are listed in the middle column.

The final performance of a deep learning model is assessed by its ability to detect objects in a new image. We prepared 10 new RAID images as a testing set to evaluate the efficiency and accuracy of the well-trained Faster R-CNN detector. As described previously, we marked the ground truth boundary boxes in these images to identify micro-RAIDs for comparison later on. Using the same computation conditions as the training phase, the MCI-frcnn detecting phase has a speed of 5.26 fps (frame per second)—how many images (frames) can be processed within a second. From predictions using the ground truth information on micro-RAIDs, we obtained the area under receiver operator characteristic value (AUROC = 0.85) (Figure 3C), and mean average precision value (mAP = 0.69) from the precision-recall (P-R) curve, indicating we have obtained a high precision classification model (Figure 3D). By manually compared boundary boxes in each pair of images of those micro-RAIDs detected by MCI-frcnn (right column in Figures 3E, 4) and their ground truth (left column in Figures 3E, 4), we found that the gIoU value (mean gIoU = 76%) could indicate the efficiency of MCI-frcnn should be enough.

The errors were then assessed as shown in Figure 3F, for all the 46 ground truth micro-RAIDs identified *via* LabelMe, and 45 of them were detected *via* MCI-frcnn, regarding as the true positive (TP) error; only 1 of them was not detected, that is, showed a false negative (FN) error. The important evaluation metric in machine learning F1 score is 99%, which indicates that we have gotten a robust algorithm of micro-domain for binary classification (yes or no). These reflect a low error level of MCI-frcnn when performing detection (Figure 4).

We then further compared the boundaries defined by MCI-frcnn with MCI-2kde, which is a two-dimensional kernel density estimation contour map-based micro-domain caller described previously (Tian et al., 2022). Specifically, the example screenshot shows highly consistent micro-RAID boundaries defined by LabelMe, MCI-2kde, and MCI-frcnn programs (Figure 3G). When the intersecting region of micro-RAIDs from MCI-2kde versus LabelMe was compared to that of micro-RAIDs from MCI-2kde versus MCI-frcnn, we obtained high correlation (Figure 3H), indicating MCI-2kde was a good method for micro-RAID boundary definition automatically and MCI-frcnn is an alternative good method for micro-RAID boundary definition. Taken together, these results confirm that micro-RAID boundaries defined by deep learning-based MCI-frcnn, were highly consistent across annotations performed by LabelMe manually and by the machine learning-based MCI-2kde method.

### Applied MCI-frcnn for micro-TAD boundary detection

We also used MCI-frcnn for micro-TAD boundary detection using human SPRITE data from the GM12878 cell line (Quinodoz et al., 2018). From the detection results, except for one tiny micro-TAD boundary that was missed, all of the five other micro-TAD boundaries were detected with high accuracy (mean gIoU = 85%) (Figure 3I). This result indicates MCI-frcnn and can also be used for detecting other types of micro-domains in addition to micro-RAIDs.

## Discussion and conclusion

MCI-frcnn is developed for micro-domain boundary detection, which adopts a deep learning-based object detection algorithm Faster R-CNN to define the boundaries of high-resolution topologically associated domains calling. MCI-frcnn includes five phases: the data preparing phase, annotation phase, training phase, detecting phase, and micro-domain genomic coordinates transforming phase. By applying MCI-frcnn to identify micro-TADs and micro-RAIDs of single-molecule chromatin interactions data generated from ChIA-Drop and SPRITE methods in *Drosophila* and humans, we approved the high performance of MCI-frcnn on micro-domain boundary detection. In addition, we demonstrated the comparability for micro-domain assessment between MCI-frcnn and MCI-2kde which is a two-dimensional kernel density estimation algorithm to identify micro-domain boundary automatically (Tin et al., 2022). MCI-frcnn offers alternative approaches of chromatin topology analysis for single-molecule chromatin interactions data.

In summary, in this work, we developed a deep learning-based Faster R-CNN detector, MCI-frcnn, for helping scientists automatically define the boundaries of micro-domains. MCI-frcnn shows high accuracy and fast speed for micro-domain boundary detection. In addition, MCI-frcnn is generalizable and can be used on source data from different techniques and species.

## Data Availability

Publicly available datasets were analyzed in this study. These data can be found at: All data used in this work are from the public dataset. ChIA-Drop data on *Drosophila melanogaster* S2 cell line are sourced from the Gene Expression Omnibus (GEO) database with the accession number GSE109355; SPRITE data on the human GM12878 cell line were obtained from GSE114242. Scripts of MCI-frcnn is available at the public repository GitHub (https://github.com/ZhengMZLab/MCI-frcnn).
