# Supplementary material for: MCI-frcnn: A deep learning method for topological micro-domain boundary detection
Source: Front Cell Dev Biol. 2022 Nov 30;10:1050769. doi: 10.3389/fcell.2022.1050769 (PMC9749004; doi:10.3389/fcell.2022.1050769)
Supplement: Supplementary file 1 [file DataSheet1.docx]

**MCI-frcnn: A Deep Learning Method for Topological Micro-Domain Boundary Detection**

Simon Zhongyuan Tian^1*^, Pengfei Yin^1^, Kai Jing^1^, Yang Yang^1^, Yewen Xu^1^, Guangyu Huang^1^, Duo Ning^1^, Melissa J. Fullwood^2,3,4*^ and Meizhen Zheng^1*^

**Supplementary materials**

**MCI-frcnn** is a *keras* (Keras: Deep Learning for humans, 2022) implementation of Faster R-CNN (Ren et al., 2015) using a TensorFlow backend (TensorFlow, 2022) for micro-domain boundary detection, which was mainly derived from the public GitHub repository *keras.frcnn* (Bardool, 2022). Input images of micro-domains for MCI-frcnn are generated by using MCI-view, the visualization module of the MCIBox toolkit (Tian et al., 2022) based on clustering algorithms to display multiplex chromatin interactions at single-molecule level. Faster R-CNN is a widely used deep learning detector for object detection (Ren et al., 2015) in computer vision field, which consists of a Region Proposal Network (RPN) module and a Fast Region-based Convolutional Neural Network (Fast R-CNN) module. MCI-frcnn adopted ResNet-50 (He et al., 2016) as the backbone for feature extraction shared by RPN and Fast R-CNN, which are the two sources of the loss value (*total_loss = RPN_loss + Fast R-CNN_loss*).

**RPN Loss** function includes a *softmax_with_loss* layer to compute binary classification loss, and a *smooth_L1_* algorithm to calculate bounding box regression loss (Ren et al., 2015), defined as follows:

| $L\left( p_{i},t_{i} \right)=\frac{1}{N_{cls}}\sum_{i} L_{cls}\left( p_{i},p_{i}^{*} \right)+\lambda\frac{1}{N_{reg}}\sum_{i} p_{i}^{*}L_{reg}\left( t_{i},t_{i}^{*} \right)$ | (1) |
| --- | --- |

where, $p_{i}$ is predicted probability of anchors contains an object or not, $p_{i}^{*}$ is ground truth value of anchors contains and object or not, $t_{i}$ is coordinates of predicted anchors, $t_{i}^{*}$ is ground truth coordinate associated with bounding boxes, $L_{cls}$ is binary classifier loss (*softmax* with *cross_entropy_loss*), $L_{reg}$ is regression loss, $L_{reg}=R\left( t_{i},t_{i}^{*} \right)$, $R$ is the *smooth_L1_* function, $N_{cls}$ is normalization parameter of mini-batch size, $N_{reg}$ is normalization parameter of regression, $\lambda$ let both $cls$ and $reg$ terms roughly equally weighted. $p_{i}^{*}L_{reg}$ means the regression loss is activated only for positive anchors. Bounding box regression tries to find the most similar anchor box for a ground truth one, whose four coordinates parametrization is shown as:

| $t_{x}=\frac{\left( x-x_{a} \right)}{w_{a}} {; t}_{y}=\frac{\left( y-y_{a} \right)}{h_{a}}; t_{w}= \log\frac{w}{w_{a}} ; t_{h}= \log\frac{h}{h_{a}};$  $t_{x}^{*}=\frac{(x^{*}-x_{a})}{w_{a}} ; t_{y}^{*}=\frac{(y^{*}-y_{a})}{h_{a}}; t_{w}^{*}= \log\frac{w^{*}}{w_{a}} ; t_{h}^{*}= \log\frac{h^{*}}{h_{a}}$ | (2) |
| --- | --- |

where $x$ and $y$ indicate the pixel location of the box center, $w$ and $h$ represent the weight and height of the bounding box, $x$ is a predicated box, $x_{a}$ is an anchor box, and $x^{*}$ indicates a ground truth bounding box.

**Fast R-CNN Detector Loss** is sourcing from a *softmax* function to identify micro-domain category versus the background, and a *smooth_L1_* function for bounding box regression for finetune (Ren et al., 2015). Fast R-CNN loss is defined as follows:

| $L\left( p,u,t^{u},t \right)=L_{cls}\left( p,u \right)+\lambda L_{loc}\left( t^{u},v \right)$ | (3) |
| --- | --- |

where, $p=(p_{0},p_{1},\ldots,p_{k})$ is a discrete probability distribution over $k+1$ outputs, $t^{u}$ is the coordinate of predicted bounding box, $v$ is the ground truth bounding box, $L_{cls}$ is the classification loss, $L_{loc}$ is the bounding box regression loss of Fast R-CNN, λ is the hyper-parameter to balance the two tasks.

**IoU, gIoU, vIoU** are coefficients used in this work to evaluate similarity between two objects (such as ground truth bounding box vs. RPN anchor box), described as following and illustrated in Figure 3A.

**IoU** (Intersection over Union) in Faster R-CNN is used for bounding box regression, which is to measure the similarity between the ground truth and the predicted bounding boxes by computing their area ration of overlapping part over their union part (Ren et al., 2015).

| $IoU=\frac{\left\vert B\cap B^{*} \right\vert}{\left\vert B\cup B^{*} \right\vert}$ | (4) |
| --- | --- |

where $B^{*}=\left( x^{*},y^{*},w^{*},h^{*} \right)$ is the ground truth bounding box, $B=\left( x,y,w,h \right)$ is the predicted bounding box, $(x,y)$ is the middle point coordinate of a box, $w and h$ are its width and height. As shown at the top left conner of Figure 3A.

**gIoU** (genomic Intersection over Union) used in this work is to calculate the ratio of genomic length (base pair) of overlapping region divided by the whole union of the two genomic regions in order to reflect the similarity between the two different regions.

| $gIoU=\frac{\left\vert R^{a}\cap R^{b} \right\vert}{\left\vert R^{a}\cup R^{b} \right\vert}$ | (5) |
| --- | --- |

where $R^{a}=\left( S^{a},E^{a} \right)$ and $R^{b}=\left( S^{b},E^{b} \right)$ are two genomic regions, $S and E$ are the start and end coordinates of the two genomic regions. As shown at the bottom left conner of Figure 3A.

**vIoU** (vertical Intersection over Union) is to detect how same of two bounding boxes occupying a same vertical region (e.g., a cluster range). By calculating the length of pixels, they overlapped along the y-axis upon their union length.

| $vIoU=\frac{\left\vert Y^{a}\cap Y^{b} \right\vert}{\left\vert Y^{a}\cup Y^{b} \right\vert}$ | (6) |
| --- | --- |

where $Y^{a}=\left( Y_{top}^{a},Y_{bottom}^{a} \right)$ and $Y^{b}=\left( Y_{top}^{b},Y_{bottom}^{b} \right)$ are y-axis coordinates of two bounding boxes. In this work we merged the detected boxes to one micro-domain if their vIoU ratio was more than 80%. As illustrated at the top right conner of Figure 3A.

**Reference**

Bardool, K. (2022). keras-frcnn. Available at: https://github.com/kbardool/Keras-frcnn [Accessed April 21, 2022].

He, K., Zhang, X., Ren, S., and Sun, J. (2016). Deep Residual Learning for Image Recognition. in *2016 IEEE Conference on Computer Vision and Pattern Recognition (CVPR)* (Las Vegas, NV, USA: IEEE), 770–778. doi: 10.1109/CVPR.2016.90.

Keras: Deep Learning for humans (2022). Available at: https://github.com/keras-team/keras [Accessed April 21, 2022].

Ren, S., He, K., Girshick, R., and Sun, J. (2015). Faster R-CNN: Towards Real-Time Object Detection with Region Proposal Networks. *IEEE Trans. Pattern Anal. Mach. Intell.* 39, 1137–1149. doi:10.1109/TPAMI.2016.2577031

TensorFlow (2022). Available at: https://tensorflow.google.cn/ [Accessed November 1, 2022].

Tian, S. Z., Li, G., Ning, D., Jing, K., Xu, Y., Yang, Y., et al. (2022). MCIBox: a toolkit for single-molecule multi-way chromatin interaction visualization and micro-domains identification. *Briefings in Bioinformatics*, bbac380. doi: 10.1093/bib/bbac380.
